# Seroprevalence of Antitransglutaminase and Antiendomysium Antibodies in Adult Colombian Blood Bank Donors

**DOI:** 10.1155/2020/7541941

**Published:** 2020-11-30

**Authors:** Sara Paredes-Echeverri, Ayda N. Rodríguez, Wilmer A. Cárdenas, Belén Mendoza de Molano, John M. González

**Affiliations:** ^1^Grupo de Ciencias Básicas Médicas, School of Medicine, Universidad de Los Andes, Bogotá, Colombia; ^2^National Blood Bank Colombian Red Cross, Bogotá, Colombia; ^3^Department of Biological Sciences, School of Sciences, Universidad de Los Andes, Bogotá, Colombia; ^4^Hospital Universitario Fundación Santa Fe de Bogotá, Gastroenterology Division, Bogotá, Colombia

## Abstract

Celiac disease (CD) is an autoimmune enteropathy induced by the ingestion of gluten from wheat, barley, and rye in genetically susceptible individuals. The global prevalence of CD is 1.4%. However, most of the prevalence studies have been conducted in Caucasian populations; few studies have been performed in Latin America. The aim of this study is to determine the seroprevalence of auto-antibodies used as markers for CD in a Colombian cohort. In this cross-sectional study, the serum samples from Colombian donors of the National Red Cross Blood Bank were collected between June and September 2017 in Bogotá, Colombia. All sera were tested for IgA antitissue transglutaminase (TTG) by enzyme-linked immunosorbent assay. Seropositive sera were tested for IgA antiendomysium (EMA) using indirect immunofluorescence assay. The ancestral genetic composition was determined in donor samples with antibody assay reactivity. Those with two seroreactive assays were typed for HLA class II DQ2 and DQ8. In total, 228 blood donors participated in the study. Among them, 113 were females (49.56%) with an average age of 31.63 years (SD ± 12.99); males had an average of 34.71 years (SD ± 13.01). Only 3 (1.31%) donors reported chronic diarrhea and nonintentional weight loss; 11 (4.82%) had a family history of CD. For the serological assays, 11 donors (4.82%) were seroreactive to IgA anti-TTG: 3 had high reactivity and 8 had low reactivity. Of those seroreactive to IgA anti-TTG, 3 (1.32%) were also seroreactive to anti-EMA, and they were typed as HLA-DQ8 or HLA-DQ2. The baseline ancestral percentage of the seroreactive donors was higher for European and Native American than for African genes. The seroprevalence for anti-TTG and anti-EMA with the presence of HLA-DQ8 and HLA-DQ2 was 1.32%. Additionally, 4.82% donor participants were reactive only for anti-TTG. Compared with other studies, our findings suggest that Colombia has a high prevalence of CD markers.

## 1. Introduction

Celiac disease (CD) is an autoimmune enteropathy with systemic manifestation that is induced by the ingestion of gluten from wheat, barley, and rye in genetically susceptible individuals. The clinical manifestations vary widely among patients; however, gastrointestinal symptoms, including chronic diarrhea, flatulence, weight loss, and abdominal pain, are the most frequent due to enterocyte damage [1]. Some patients with CD also present with extraintestinal involvement, including dermatological, neurological, gynecological, and musculoskeletal manifestations [2–6]. When gliadin, a protein derived from gluten, is in the small intestine lumen, and it triggers the zonulin-dependent increase of gut permeability and activates an innate immune response. Then, the deaminated form of gliadin initiates an adaptative immune response [7, 8]. This immunological cascade leads to chronic inflammation and the presence of autoantibodies against tissue transglutaminase (TTG), endomysium (EMA), and deaminated gliadin [9].The human genetic predisposition for CD has been well studied, and the presence of HLA class II alleles, HLA-DQ2 or HLA-DQ8, is considered a risk factor [10]. The first diagnostic criteria for CD were published in 1970 [11] and had been updated by the American College of Gastroenterology, British Society of Gastroenterology, and NICE guidelines. The current criteria include the following: (I) intestinal and extraintestinal symptoms; (II) genetic markers HLA-DQ2 or HLA-DQ8; (III) normal total serum IgA levels in conjunction with the presence of autoantibody IgA anti-EMA or IgA anti-TTG; and (IV) histological findings that vary from lymphocytic infiltrate to villous atrophy [12–14]. In 2020, the European Society for Pediatric Gastroenterology, Hepatology, and Nutrition (ESPGHAN) drafted the diagnostic criteria for children sparing intestinal biopsies if the serological and genotypic markers are present [15, 16].

The prevalence and seroprevalence of CD have been evaluated using a diverse set of serological markers. In general, the prevalence is estimated with findings on intestinal biopsy in conjunction with the presence of IgA anti-EMA and anti-TTG autoantibodies in sera [17]. The pooled global seroprevalence for anti-TTG and/or anti-EMA is 1.4%; however, most studies have been performed in predominantly Caucasian populations [17]. In Latin America, few studies have estimated the seroprevalence and burden of the disease. In a review of the literature, Brazil, Argentina, and Mexico have reported seroprevalence up to 0.95% [18–20], 2.70% [21], and 2.67% [22], respectively. Pooling the seroprevalence is difficult because studies test different antigen-specific autoantibodies and isotypes. Among these serological tests, the most sensitive is the detection of IgA anti-TTG antibodies by ELISA, and the most specific test is the detection of IgA anti-EMA by immunofluorescence [23]. It is recommended to test first for IgA anti-TTG, and then, if the result is at least a weak positive, test for IgA anti-EMA [7].

Due to the lack of information on CD prevalence in samples with mixed genetic descent [24], the goal of this study was to determine the seroprevalence of CD antibodies in Colombian blood donors. It is hypothesized that due to the multiracial population, Colombians would have less burden of disease compared to European countries, but comparable to other Latin American populations. In this study, serological assays to detect anti-IgA for TTG by ELISA and IgA anti-EMA by indirect immunofluorescence assay were carried out, and genetic testing was performed in seropositive samples.

## 2. Materials and Methods

### 2.1. Study Population and Sampling

Blood samples were withdrawn from 228 adult donors of the National Blood Bank of the Colombian Red Cross in Bogotá between June and September 2017. Each donor completed a symptom questionnaire and signed an informed consent form. Two samples were taken by venipuncture: one without any anticoagulant and another with EDTA in vacutainer tubes (BD Biosciences, San Jose, California, USA). Whole-blood samples were centrifuged at 1,350 g for 5 min, and serum was obtained, aliquoted, and stored at −20°C for antibody testing. EDTA tubes were stored at −70°C for DNA extraction, HLA typing. and ancestry testing. The protocol and informed consent were done according to the Colombian regulation (Resolution 8430 from 1993) and the guidelines of the Declaration of Helsinki. The study and informed consent were approved by the Ethical Committees of Universidad de los Andes (Act 693, 2017) and Ethical and Research Committee from Hospital Universitario Fundación Santa Fe de Bogotá (Act 7, 2017). To maintain anonymity, each donor was assigned a three-digit number preceded by the acronym SEC (Celiac Disease Study in Spanish).

### 2.2. ELISA Using Anti-TTG Antibodies

All serum samples were tested for IgA anti-TTG using QUANTA Lite(R)h-tTG IgA ELISA (Cat. 708760, Lot. no. 037226; Inova Diagnostics, San Diego, CA, USA). The samples were diluted 1 : 100 in sample diluent. Prediluted serum was placed in each microplate well along with the negative, low-positive, and high-positive controls and was incubated for 30 min at RT. After washing, 100 *μ*L of the IgA peroxidase conjugate was added to each well and incubated for 30 min at RT. After another wash, 100 *μ*L of the TMB chromogen was added to each well and allowed to incubate in the dark for 30 min. Next, 100 *μ*L of the stop solution was added to each well, and then the absorbance was read in a spectrophotometer (Bio-Rad model 680, Hercules, CA, USA) at a 450 nm wavelength.

### 2.3. Indirect Immunofluorescence Assay Using Anti-EMA Antibodies

ELISA IgA anti-TTG seroreactive samples (11 donors, Table 1) were tested for IgA anti-EMA using the AESKUSLIDES-EMA kit (Ref. 512.050, Lot. no. 18044; AESKU Diagnostics, Wendelsheim, Germany). The slides were placed in a humid chamber for 30 min. The donor serum was diluted 1 : 5, placed in the well, and incubated for 30 min along with the positive and negative controls. After washing the slides, 50 *μ*L of IgA conjugated with the FITC fluorochrome was added to each well, and the samples were then incubated for 30 min in the dark followed by washing with 1x PBS. Evans blue dye diluted at 1 : 3000 was added to each well (Sigma-Aldrich, St. Louis, MI, USA), the slides were washed and allowed to dry, and then mounting medium (Gelvatol; Sigma-Aldrich, St. Louis, MO, USA) was added to each slide. The slides were analyzed under a fluorescence microscope by two independent readers (Zeiss, Oberkochen, Germany).

### 2.4. HLA Class II Typing Assay

DNA from the three donors with both seroreactive assays was extracted using the Abbott m2000 RealTime automated DNA extraction kit (Abbott Park, IL, USA). The typing of the class II HLA alleles for DQA and DQB was performed using single-strand PCR and the commercial kit Immucor-Lifecodes Inc. (Norcross, Georgia, USA) followed by analysis using the Luminex 200 system (R&D Systems, Minneapolis, MN, USA).

### 2.5. Ancestral Genetic Composition

Seroreactive samples were genotyped for 46 ancestry-informative markers (AIM-Indel markers) distributed across the autosomal genome. PCRs were carried out in a single multiplex containing all 46 primer pairs according to the protocol described by Pereira et al. [25].

### 2.6. Result Interpretation and Data Analysis

ELISA to detect anti-TTG was interpreted according to the optic densities (ODs) of each well according to the manufacturer's instructions: units of the sample = (average OD of the sample/OD of the low-positive control) *∗* units of the low positive are specified on the label. Units ≥30 were considered high positives, 20–29 low positives, and <20 negatives. The immunofluorescence slides to detect anti-EMA were interpreted blindly by two independent researchers, and the outcome was binary: either reactive or not reactive. HLA class II PCR typing resulted in gene description, and the ancestral study was presented as a percentage of pedigree dominance.

The data obtained were represented as descriptive analysis with means, standard deviations, and comparisons within the age group and sex.

## 3. Results

The study included 228 participants with an average age of 33.18 years (SD ± 13.06) and an age range from 18 to 65 years. The distribution by age is shown in Supplementary Figure 1. Of these, 113 were females (49.56%) and had an average age of 31.63 years (SD ± 12.99); the average age of males was 34.71 years (SD ± 13.01).

Only 8 participants (3.50%) referred that they did not consume gliadin-containing products without indicating any specific reason, 3 (1.31%) had a medical history of having more than 1 week of diarrhea, and another 3 (1.31%) referred nonintentional weight loss. From the total, 11 (4.82%) participants had a family member who was being studied for CD or followed a gluten-free diet. Interestingly, none of the individuals with reactive serology reported any of the aforementioned symptoms or family history, and they all claimed to be asymptomatic.

**Figure 1:**
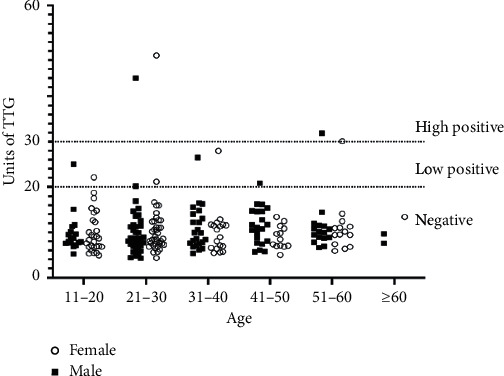
Distribution of seroreactive individuals according to age and sex. *X*-axis: donors were divided according to age distribution and sex. *Y*-axis: units of IgA anti-TTG according to reactivity: units ≥30 were considered high positives, 20–29 low positives, and <20 negatives.

From the donors, 11 (4.82%) were seroreactive for IgA anti-TTG: 3 donors had high reactivity and 8 donors had low reactivity. The sex and age of seropositive IgA anti-TTG donors (5 females and 6 males) did not correlate with the reactivity of the test (Figure 1).

Of those seropositive for IgA anti-TTG, 3 (1.32%) were also seropositive for IgA anti-EMA, and all three were typed HLA-DQ8 or HLA-DQ2 (Table 1).

**Figure 2:**
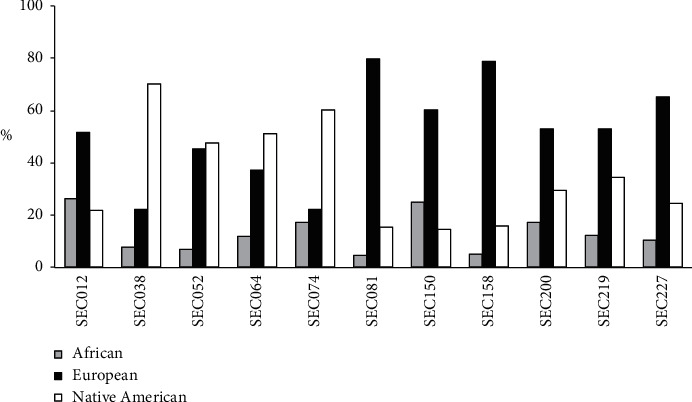
Percentages of ancestries in donors with seroreactive assays. African, European, and Native American membership percentages in samples from donors with seroreactive antibody assays.

Seven donors had a higher baseline European ancestry, including donors SEC081 and SEC227 (80% and 65%, respectively), who were reactive to both antibodies, whereas four donors had higher Native American ancestry, including donor SEC038 (70%), who was also reactive to both antibodies. The percentage of the ancestral membership of each seropositive donor is represented in Figure 2.

## 4. Discussion

In this study, CD seroprevalence was determined in samples from Colombian blood donors. Additionally, an ancestry study was performed in donors reactive to IgA anti-TTG and in those who were reactive to both IgA anti-TTG and anti-EMA, typed for HLA class II DQ.

Currently, there is a worldwide tendency toward an increase in the diagnosis of CD either because of an increment in burden or a growing awareness of the disease [7]. Historically, this disease was considered to be more restricted to the Caucasian population, but the gain of prevalence studies has proven that CD is present worldwide [17]. There is a difference between the global seroprevalence rate of anti-TTG and/or anti-EMA (1.4%) and the biopsy-confirmed prevalence (0.70%) due to a high rate of possible false-reactive serology test [17].

In Latin America, few studies have estimated the prevalence of CD [18–22, 26–34]. The largest epidemiological studies have been performed in Mexico, Argentina, and Brazil, and there is a difference between the reported CD prevalence and seroprevalence ranging from 0 to 3.03% (Table 2). Seroprevalence changes could be associated with the differences between the study populations, lack of consistency in the outcome measurements, and changes in diagnostic guidelines. In Colombia, only one other study has measured CD antibodies in healthy adults [28]. In that study, out of the 140 donors in the control group living in the northwest region of the country, 5 had IgA antigliadin, 2 had IgG antigliadin, and no one had IgA or IgG anti-TTG; and of the 120 control donors living in the central region of the country, none tested positive for serological CD markers [28]. From studies conducted in blood bank donors, in two Brazilian studies, they found a seroprevalence of 0.28–0.60% [18, 26], while in Iceland, it was 0.74% [35]. Our study found that the estimated seroprevalence only for IgA anti-TTG was 4.82%, and for IgA anti-TTG and anti-EMA with the presence of HLA-DQ8 and HLA-DQ2, it was 1.32%. Although this is high but consistent with the estimated global seroprevalence of CD markers [17], it is possible that our results can be overestimating the true prevalence of the disease.

Interestingly, it is well known that CD is more prevalent in females, and there is an increasing prevalence of the disease with age in the adult population [17]. Our study did not find sex or age differences between seroreactive and non-seroreactive donors, probably because our study only assessed serological markers, the complete criteria of CD diagnosis were not performed, and the cohort was small.

The three donors that were reactive to both IgA anti-TTG and anti-EMA had the genetic risk factor HLA-DQ8 or HLA-DQ2. Interestingly, two of these donors have a higher European ancestry. However, SEC038 had a higher Native American ancestry. Ossa et al. found that, on average, individuals from this area of the country are 25% Native Americans and 67% Europeans [24].

## 5. Conclusions

The main limitation of this study is a small sample size. Also, for future studies, we could measure the total IgA levels in serum since IgA deficiency is prevalent in CD, and these patients have an increased likelihood of having false-negative assay results [7]. Our findings suggest that Colombia has a comparable seroprevalence of CD markers to the rest of the world. Follow-up studies are needed to extrapolate the prevalence of CD in the Colombian population. However, this study provides the first insight of the epidemiologic trends of CD in the country.

## Figures and Tables

**Table 1:** Summary of seroreactive donors.

Donor	Sex	Age	ELISA		IFAT	PCR
			Transglutaminase		Endomysium	HLA alleles
			Reactivity	Units^†^	Reactivity	
SEC012	Male	36	Low	26.47	−	ND
SEC038	Female	36	Low	27.97	+	DQB1*∗*0302^††^
SEC052	Female	23	High	49.01	−	ND
SEC064	Female	23	Low	21.14	−	ND
SEC074	Male	29	High	43.98	−	ND
SEC081	Female	55	Low	30.00	+	DQB1*∗*0302^††^
SEC150	Male	20	Low	25.03	−	ND
SEC158	Female	18	Low	22.07	−	ND
SEC200	Male	23	Low	20.19	−	ND
SEC219	Male	52	High	31.87	−	ND
SEC227	Male	46	Low	20.80	+	DQA1*∗*0201: DQB1*∗*0202^†††^

ELISA: enzyme-linked immunosorbent assay; IFAT: indirect fluorescent antibody test; PCR: polymerase chain reaction; HLA: human leukocyte antigen; ND: not done. ^†^Units: (average optical densities of the sample/optical densities of low positive) × units of TTG low positive (specified on the label). ^††^DQ8: corresponds to the molecular alleles DQB1*∗*0302 or DQB1*∗*0305. ^†††^DQ2: corresponds to the molecular alleles DQB1*∗*0201, DQB1*∗*0202, or DQB1*∗*0203. The alleles DQA1*∗*0201: DQB1*∗*0202 and DQA1*∗*0501: DQB1*∗*0201 are termed DQ2.2.

**Table 2:** Celiac disease prevalence studies performed in Latin America.

Study	Ref	Country	No.	Gliadin		TTG		EMA		Biopsy	Seroprevalence (%)	Prevalence (%)
				IgA	IgG	IgA	IgG	IgA	IgG			
Gomez et al., 2001	[30]	Argentina	2,000	+	+	−	−	+	+	+	0.50	0.59
Mora et al., 2010^*∗*^	[31]	Argentina	2,219	−	−	+	−	+	−	+	1.30	1.26
Vázquez et al., 2015^*∗∗*^	[21]	Argentina	144	−	+	+	−	+	−	−	2.70	NP
Alencar et al., 2012	[18]	Brazil	4,000	−	−	+	−	+	−	+	0.60	0.35
Almeida et al., 2013^*∗∗∗*^	[19]	Brazil	946	−	−	+	−	+	−	−	0.95	NP
Almeida et al., 2012^*∗∗∗∗*^	[20]	Brazil	840	−	−	−	−	+	−	−	0.00	NP
Gandolfi et al., 2000	[32]	Brazil	2,045	+	+	−	−	+	+	+	3.03	0.15
Melo et al., 2006	[33]	Brazil	3,000	−	−	+	−	+	−	+	0.50	0.33
Oliveira et al., 2007	[34]	Brazil	3,000	−	−	+	−	−	−	+	1.50	0.47
Pereira et al., 2006	[26]	Brazil	2,086	−	−	+	−	+	−	+	0.28	0.24
Utiyama et al., 2010^*∗∗*^	[27]	Brazil	321	−	−	−	−	+	+	−	0.00	NP
Parra-Medina et al., 2015	[28]	Colombia	260	G1	G1	G1, G2	G1	G2	−	−	2.69	NP
Galván et al., 2009	[29]	Cuba	200	−	−	+	+	−	−	+	0.50	0.50
Remes-Troche et al., 2006	[22]	Mexico	1,009	−	−	+	−	−	−	−	2.67	NP

Ref: reference; No.: study population; gliadin: antigliadin antibody testing; TTG: anti-TTG antibody testing; EMA: anti-EMA antibody testing; G1: samples from the northwestern region of Colombia; G2: samples from the central area of Colombia. (+): done; (−): not done; NP: not provided. ^*∗*^The samples were from children; ^*∗∗*^the samples were from the Amerindian population; ^*∗∗∗*^the samples were from the elderly; ^*∗∗∗∗*^the samples were from the Afro-descendant population.

## Data Availability

The data used to support the findings of this study are restricted by the Ethics Committee of Fundación Santa Fe de Bogotá to protect patient privacy. The data are available from John M. González at johgonza@uniandes.edu.co for researchers who meet the criteria for access to confidential data.

## Supplementary Materials

Supplementary Materials Supplementary Figure 1: age distribution of blood donors.
