# Supplementary material for: Seroprevalence of Antitransglutaminase and Antiendomysium Antibodies in Adult Colombian Blood Bank Donors
Source: Can J Gastroenterol Hepatol. 2020 Nov 30;2020:7541941. doi: 10.1155/2020/7541941 (PMC7723479; doi:10.1155/2020/7541941)
Supplement: Supplementary Materials — Supplementary Figure 1: age distribution of blood donors. [file 7541941.f1.docx]

**Supplementary Figure**

Supplementary Figure 1. *Age distribution of blood donors.*
